# Infection Frequency of Hepatitis C Virus and IL28B Haplotypes in Papua New Guinea, Fiji, and Kiribati

**DOI:** 10.1371/journal.pone.0066749

**Published:** 2013-08-20

**Authors:** G. L. Abby Harrison, Jan Pryor, Joji Malani, Mathias Supuri, Andrew Masta, Burentau Teriboriki, Tebuka Toatu, David Penny, Jean-Pierre Allain, Eleanor Barnes, Oliver G. Pybus, Paul Klenerman

**Affiliations:** 1 Peter Medawar Building for Pathogen Research, Nuffield Department of Medicine, University of Oxford, Oxford, United Kingdom; 2 Walter+Eliza Hall Institute for Medical Research, Melbourne, Victoria, Australia; 3 Department of Medical Science, Fiji National University, Suva, The Republic of Fiji; 4 Obstetrics and Gynaecology, Pacific International Hospital, Port Moresby, National Capital District, Papua New Guinea; 5 School of Medicine and Health Sciences, University of Papua New Guinea, Boroko, Port Moresby, National Capital District, Papua New Guinea; 6 Tabiteuea Hospital, Kiribati Ministry of Health, Tabiteuea, Kiribati; 7 Disease Surveillance, Control and Research Unit, Secretariat of the Pacific Community, Suva, The Republic of the Fiji; 8 Institute of Fundamental Sciences, Massey University, Palmerston North, New Zealand; 9 National Blood Service East Anglia Blood Centre and Division of Transfusion Medicine, Department of Haematology, University of Cambridge, Cambridge, United Kingdom; 10 Nuffield Department of Medicine, the Oxford National Institute for Health Research Biomedical Research Centre and the Oxford Martin School, Oxford University, Oxford, United Kingdom; 11 Department of Zoology, University of Oxford, Oxford, United Kingdom; Centers for Disease Control and Prevention, United States of America

## Abstract

It has been estimated that there are more than 60 million Hepatitis C virus (HCV) carriers in the World Health Organisation's Western Pacific region (WHO-WPR), where liver cancer is among the top three causes of cancer death. WHO and the US Centres for Disease Control and Prevention report the prevalence of HCV in the South Pacific islands (countries within the WHO-WPR) to be high (5–10% and >2% respectively). However, since HCV is not tested for in many of these countries, there is sparse data available to support this assertion. We screened ∼2000 apparently healthy individuals from Papua New Guinea, Fiji and Kiribati and found a sero-prevalence of 2.0%, 0.1% and 0%, respectively. All sero-positive samples tested negative for HCV RNA. Curious as to why all the sero-positive individuals were negative for HCV-RNA, we also screened them for the HCV protective IL28B SNP markers rs12979860 and rs8099917. All antibody-positive participants bar one had HCV protective haplotypes. Our results suggest that HCV is present in these Pacific island countries, albeit at a prevalence lower than previous estimates. As none of our participants had undergone antiviral treatment, and therefore must have cleared infection naturally, we hypothesise that genotypes 1 and/or 4 are circulating in South Pacific Island people and that these peoples are genetically predisposed to be more likely to spontaneous resolve HCV infection than to become chronic carriers.

## Introduction

Isolated in 1989, the hepatitis C virus (HCV) has since been found to be a globally diverse, positive sense, RNA virus, belonging to the Flaviviridae family, and is classified into at least 7 genotypes and numerous subtypes [1]–[4]. Each genotype, and subtype, displays different patterns of endemic and epidemic epidemiology. For example, subtype strains such as 1a, 1b, 2a, 2b, 3a, and 4a, are linked with 20th century outbreaks caused by the use of unscreened blood products, illicit drug injecting, or non-sterile medical injections [5]–[10]. In contrast, other local endemic strains appear to have been present in particular populations for many centuries, such as genotypes 1 and 2 in West Africa and genotype 6 in South East Asia. Genotype 6, for example, exhibits a very high degree of genetic diversity, and evolutionary molecular clock analyses suggest that the subtypes of this genotype diverged at least 1000 years before present [6].

HCV exposure in 50–80% of cases (depending on host and virus factors) [11]–[14] leads to a chronic infection that can in turn result in chronic liver disease, liver cirrhosis and liver cancer, each resulting in considerable morbidity and mortality. The high prevalence of HCV infection makes it a health problem of international importance. Studies over the last 23 years have identified that clinical outcomes depend on both host and viral factors. For example, individuals infected with genotype 1 are less likely to clear viral infection upon treatment [15]. Recent genome-wide association studies findings have identified haplotypes of single nucleotide polymorphic markers (SNPs) strongly associated with spontaneous and treatment-induced clearance in genotypes 1 and 4 (i.e., the C in haplotype rs12979680, and the T in haplotype rs8099917) [16]–[18].

The primary risk factor for HCV infection is exposure to infected blood or blood products, for example, via unscreened blood transfusions or cutaneous injury with HCV contaminated needles, piercing instruments, tattoo pens, or surgical and dental instruments. Throughout the 21^st^ century the causes of local epidemics have included unsafe medical practices, illicit injecting drug users (IDUs), and unsafe vaccination practises. Whilst rare, HCV can also be transmitted via sexual exposure. HCV has also been detected in saliva, breast milk, urine, and faeces, as well as semen and cervico-vaginal secretions [10].

Any effort to prevent transmission and control HCV requires accurate epidemiological data. Serological screening assays have played a major role in understanding HCV epidemiology and have reduced the risk of HCV transmission and the global disease burden considerably. Unfortunately there is much debate with regards to their most efficacious usage and interpretation [19]–[24]. The many tests vary in cost, sensitivity, and efficiency resulting in a plethora of recommendations on appropriate use and interpretation. The implementation of efficacious screening programs in the developing South Pacific Island Countries (SPICs) is primarily hindered by cost, but is also problematic because these assays commonly produce biological false-positive reactions, particularly in populations of low HCV prevalence and in tropical environments where the frequent level of exposure to other pathogens (such as malaria and dengue virus) can cause cross reactivity [11], [25]–[28]. Nonetheless, there have been a few HCV prevalence surveys in SPICs; the earlier screening surveys (from serum samples collected as early as 1984, but predominantly from 1990–91) presented anti-HCV sero-prevalence rates up to 8% [29]–[34]. More recent studies, circa 2006, have estimated lower infection rates of 2–4% [35]. Despite HCV being endemic world-wide and despite there being strong indicators of HCV presence in SPICs, the true epidemiology of HCV infection in the developing nations of the 22 SPICs is not known, as the resources for high-quality studies are not readily available and national screening procedures are not in place.

Here we estimate the frequency of HCV sero-reactivity and RNA carriage in three SPICs Papua New Guinea (PNG), Fiji and Kiribati, amongst otherwise healthy adults. These three low-to-middle income nations (as classified by the World Bank) carry a high burden of Hepatitis B Virus (HBV) [36], [37]. The use of marijuana and alcohol is widespread in these Pacific Island societies and is closely associated with unprotected sexual activity and acts of sexual violence, however, injecting drug use is not recognised to be prevalent in the region. The incidence of injecting drug abuse is considered to be extremely rare (pers. comm. Dr Kelly, PNG Institute of Medical Research); it has only been recorded as the mode of transmission in one case of HIV transmission. Interestingly tattooing is also not considered to be an important risk factor [39], it remains to be seen if penile injecting, which is wide spread in the region, is similarly not associated. Fiji introduced HCV serological screening for transfused blood in 2006 but neither PNG nor Kiribati screen, therefore in these countries exposure via unscreened blood transfusion remains the primary risk factor for HCV transmission.

## Results

Table 1 provides a summary of the results of the serological assays by age, sex and country, Table 2 provides a summary of the results by country alone, and Table 3 gives the results by town within PNG. Table S1 presents the serological results including the signal-to-cut-off (S/CO) ratios, the results of the nucleic acid testing (NATs) and the IL28B results. Fourteen PNG, one Fijian (an adult female) and zero I-Kiribati samples tested reactive for both the Ortho HCV3 assay and the anti-HCV Monolisa assay.

**Table 1:** Serological HCV results by age.

	Kiribati						Fiji						Papua New Guinea					
	Male		Female		Total		Male		Female		Total		Male		Female		Total	
Age yrs	sampled	+ve	sampled	+ve	sampled	+ve	sampled	+ve	Sampled	+ve	Sampled	+ve	sampled	+ve	sampled	+ve	sampled	+ve
**0–9**	2	0	3	0	5	0	3	0	2	0	5	0	8	0	11	0	19	0
**10–19**	3	0	10	0	13	0	2	0	17	0	19	0	10	0	29	0	39	0
**20–29**	51	0	202	0	253	0	48	0	318	1	366	1	66	2	177	5	243	7
**30–39**	31	0	233	0	264	0	58	0	213	0	271	0	71	2	137	2	208	4
**40–49**	25	0	66	0	91	0	53	0	71	0	124	0	51	2	45	1	96	3
**50–59**	9	0	7	0	16	0	36	0	27	0	63	0	14	0	13	0	27	0
**60–69**	3	0	4	0	7	0	16	0	7	0	23	0	4	0	3	0	7	0
**70–79**	2	0	2	0	4	0	7	0	3	0	10	0	1	0	3	0	4	0
**80–89**	0	0	0	0	0	0	1	0	0	0	1	0	0	0	0	0	0	0
**U/N**	2	0	2	0	4	0	9	0	13	0	22	0	0	0	0	0	1*	0
**Totals**	128	0	529	0	**657**	**0**	233	0	671	1	**904**	**1**	225	6	418	8	**644**	**14**

Abbreviations: yrs, years; sampled, number sampled; +ve, number of serologically positive samples;

* , participants sex was not recorded; U/N, age not recorded.

**Table 2:** Serological HCV results by country.

	Sample size	Number seropositive	Prevalence (maximum likelihood estimate)	Prevalence (95% lower CI)	Prevalence (95% upper CI)
Kiribati	657	0	0%	0%	0.5%
Fiji	904	1	0.1%	<0.01%	0.7%
PNG	644	14	2.2%	1.3%	3.6%

Abbreviations: %, percentage; CI, Confidence Interval.

**Table 3:** Serological HCV results by town in Papua New Guinea.

Papua New Guinea					
Town	Sample size	Number seropositive	Prevalence (maximum likelihood estimate)	Prevalence (95% lower CI)	Prevalence (95% upper CI)
Port Moresby	322	7	2.2%	1.00%	4.5%
Daru	91	0	0%	0%	3.5%
Madang	73	7	9.6%	4.5%	18.7%
Goroka	59	0	0%	0%	5.3%
Mt Hagen	99	0	0%	0%	3.2%
Total	644	14	2.2%	1.3%	3.6%

Abbreviations: %, percentage; CI, Confidence Interval.

In PNG there were considerable provincial differences in HCV sero-reactivity. Samples were collected at sites representing 5 of the 19 provincial regions in PNG: Central Province (Port Moresby), Western Province (Daru), Madang Province (Madang), Eastern Highlands Province (Goroka), and Western Highlands Province (Mt Hagen). All samples collected from Daru, Goroka, and Mt Hagen were negative for HCV; 2.2% and 9.6% of the samples collected in Port Moresby and Madang respectively, tested positive. We found no significant difference in prevalence between men and women (2-tailed Fisher's Exact Test) nor between those <50 and those >50 years old (2-tailed Fisher's Exact Test). S/CO ratios (see Methods) from both assays were calculated and compared and we found inconsistent results between the assays. Five samples tested with the Ortho 3 assay consistently had S/CO ratios greater than ≥3.8 (samples P_047, P_130, P_242, PW018 and PW064) but only two tested with the Monolisa assay had a ratio ≥5 (samples P_109 and P_130). This lack of consistency reaffirms the difficulty in scoring HCV-positive samples in a resource-poor setting where the more specific recombinant immunoblot assay (RIBA) tests or HCV-NAT testing may not be available.

Duplicate NATs for HCV RNA were conducted on all samples that gave a reactive serologic screening response. No viral RNA was detectable in any sample. As part of the original HBV study we were required to return to the collection sites and provide follow-up support and feedback from the study. On our last visit in 2008, three of the participants that screened reactive for HCV returned for follow up; at this later time they were re-bled and retested and none were HCV RNA positive.

The IL28B haplotype results are listed in Table S1. There was considerable homogeneity in the populations sampled. All but one of the fifteen sero-positive samples were homozygous for the major allele C in haplotype rs12979860 and all were homozygous for the major allele T in rs8099917. The remaining sample, P_242, was heterozygous for the rs12979860 SNP. The CC/TT genotype is strongly associated with spontaneous and treatment-induced clearance in genotypes 1 and 4. [16], [17], [38]. A further eighty-eight individuals from the same three countries were typed for the rs8099917 SNP as part of another study and the results were also homogeneous (see Table S2).

## Discussion

Our results demonstrate a national HCV sero-prevalence of 2.2% in PNG, 0.1% in Fiji, and 0% in Kiribati, considerably less than previously reported but in line with the few published reports of the sero-prevalence in the SPICs [33], [35], [39]. However, we observed considerable variability in HCV sero-prevalence among the sample sites. Five sites were screened in PNG but HCV reactivity was found in only two locations (Port Moresby and Madang). These two towns are in lowland coastal areas with moderately well-supported hospitals and infrastructure, both have a notable influx of foreign workers and tourists, and both are towns that were established early in PNG colonial history. Any one of these factors may have introduced the virus to these particular provinces. HCV is unlikely to have become established by IDUs. A handful of IDUs from PNG have been reported in behavioural survey studies, but there is no known IDU cohort or culture, nor reliable estimates of IDU prevalence in PNG [40]). It is unlikely that the 9.6% sero-prevalence we found in Madang reflects the general population exposure, as closer inspection revealed that the sample population was enriched for high-risk exposure participants such as health-care workers or people that handled blood as part of their profession (e.g. nurses or phlebotomists). Indeed, individuals PW013, PW017, PW023, PW062, PW064, and PGM016 were all health-care workers of some kind. The possibility that HCV exposure might be so prevalent amongst health-care workers highlights the need for better awareness and control.

Interestingly, in concordance with all previously published studies from SPICs, no HCV RNA could be confirmed in any of the samples [29], [30], [32], [35]. For our initial samples collected in 2005, inappropriate long-term storage could be a reason for the lack of detection of HCV-RNA. However, for the three individuals retested in 2008 this is not the case as each sample was tested for HCV-RNA within an hour of the blood samples being drawn. Neither HCV screening nor treatment are available in PNG and none of the HCV-positive individuals indicated in their initial interview that they were aware of having had HCV. Therefore the infected individuals will most likely have spontaneously cleared the infection. Spontaneous clearance estimates for HCV range from 15–50% depending on the population being sampled [11], [14], [41]. Clearance has been attributed to various factors, including host innate and adaptive immunity as well as being associated with particular genetic markers such as the Il28 SNPs [14], [17], [18]. We screened all our HCV antibody-positive samples for the rs12979860 and rs8099917 SNPs; all but one individual was homozygous for the “protective” C and T haplotypes respectively. Thus a high rate of HCV clearance in these individuals may have been enhanced by the presence of these favourable IL28B alleles - it is plausible that the individuals who were HCV sero-positive but PCR negative may have had only a transient infection that spontaneously resolved. Previous work by Thomas et al. 2009 [17] who screened for the rs12979860 SNP, and by Shebl et al. 2011 [42] who screened for rs12979860 and rs8099917 markers, and a separate parallel study of our own (where eighty-eight individuals from the same three countries where screened for the rs8099917 SNP; see Table S1) have demonstrated a 70–100% prevalence of the major ‘protective’ rs12979860 and rs8099917 haplotypes in indigenous SPIC populations. However, these haplotypes are only associated with spontaneous viral clearance of genotypes 1 and 4 and no information is available regarding the circulating genotypes in these populations. If the HCV strain circulating in PNG is an ancestral endemic strain then it is more likely to be genotype 6 (the endemic strain circulating in South East Asia) or perhaps a close relation. If HCV was recently introduced it is more likely to be one of the epidemic strains such as subtypes 1a, 1b, 2a, 2b or 3a [6].

The prevalence and preponderance of HCV makes it a global health problem and accurate epidemiological data must underpin any effort to prevent transmission and control the virus. Unfortunately the epidemiology of HCV infection in SPICs is not well characterised [29]–[35]. National blood transfusion services exist in the region, to ensure a safe and secure supply as well as use of blood and blood products but only a few SPICs screen for HCV. There is no information regarding circulating genotypes. In SPICs, IDUs are extremely rare, and to date scarification and tattooing has shown no association with HCV [36]. Consequently, blood transfusions very likely present the greatest risk of infection. Clearly the primary risk factor for HCV infection (i.e., exposure to infected blood or blood products) remains neglected. We were fortunate in having a sample bank of sera collected from mother-child pairs and healthy adults in PNG, Kiribati and Fiji that were collected for the purpose of viral hepatitis research. From this bank, 2224 serum samples were made available for screening and have contributed significantly to the knowledge of HCV in the region.

Finally, there is much debate over the correct interpretation of serological test results, particularly in a resource-poor setting where RIBA testing or HCV-NAT testing may not be available. In our study five samples scored “truly positive” in the Ortho-3 assay and two in the BioRad assay, but only one sample scored “truly positive” in both assays. This reaffirms the difficulty in scoring HCV positive samples. In our work we chose to follow recommendations from the WHO and Owusu-Ofori et al 2005 [23] and scored samples as either past, or presently positive if positive in two assays. We find this to be more practical than using the S/CO ratios.

This study has revealed that the HCV sero-prevalence rate in SPICs is variable but generally lower than previously estimated. We were unable to detect HCV RNA and define genotypes in this extensive survey. The ability to assess accurately the burden of HCV disease is clearly important in defining public health priorities, as well as, indicating the history and the likely future impact of this infection. The high rate of false-positives using a single screening test suggests that caution must be applied in interpreting such assays, particularly in low-prevalence settings where there is a high burden of other chronic infections. Applying such a cautious multi-layered approach will be of value in determining the impact of HCV endemic and epidemic strains in diverse global populations.

Finally, we note that, despite the relatively low frequency of HCV, the rate of liver cancer in this region is still very high. The simplest explanation for this is the high prevalence of Hepatitis B virus infection [31].

## Materials and Methods

### Sample Collection

A collection of serum samples from PNG, Fiji and Kiribati was obtained during a study of the molecular epidemiology and evolution of HBV in the region. 1500 mother and child sample pairs and 1000 adult serum samples were collected between 2003 and 2005. Ethical permission for the blood sample collection and subsequent HCV research was obtained from each country through the appropriate national committees and/or Ministries of Health. In PNG and Fiji, permission was obtained via the PNG National Medical Research Advisory Committee and the Fiji National Research Ethics Review Committee, respectively. In Kiribati there was no established ethical review committee at the time of the project, therefore extensive consultation with the Ministry of Health and local medical professionals was conducted before the project began. Signed informed consent was obtained from each participant. Although the primary purpose of the study focused on the hepatitis B virus in the Pacific, informed participant permission was also granted to screen for HCV. In all three countries convenience group sampling was applied. In PNG blood samples were collected from participants present at outpatient clinics at regional hospitals in Port Moresby, Goroka, Mt Hagen, Madang, and Daru. These participants were either escorting family members or friends to the clinic or came specifically to be tested for HBV. Approximately 314 from Port Moresby were volunteer parents attending their children at the childrens' ward at Port Moresby General Hospital. All blood samples from Fiji were collected in Suva from the Colonial War Memorial Hospital either from the outpatients' clinic or from parents attending their children in the childrens' ward as above. In Kiribati samples were collected from parents at village preschools and from vaccination clinics on North Tarawa and North Tabiteuea. The samples included people from each of the 19 provinces of PNG, the various ethnic groups living in Fiji (Indigenous-Fijian, Indo-Fijian, Rotuman, and other Pacific Islanders) and each of the 12 Gilbert atolls of Kiribati. Blood was collected by venupuncture and serum was separated and stored at −20°C. Our sampling was targeted at adults in the community or mother-child pairs, but no specific inclusion/exclusion criteria were applied. Anyone who wished to be tested was tested. On occasions when a patient presented with hepatitis-like illness (e.g. jaundice) they were tactfully approached (if deemed healthy enough to provide informed consent by their attending physician). However there were only three such patients; none were HCV positive and 2 were HBV positive. Questionnaires were completed for each blood donor providing information on (amongst other things) age, sex, symptoms of liver dysfunction, blood transfusions, occupation and tattoos. From this set, 2224 serum samples were selected and screened for HCV.

### Serology

There are a plethora of guidelines for the use of HCV enzyme immunoassays (EIA). We chose to incorporate guidelines from WHO, Atlanta CDC, and Owusu-Ofori et al 2005 [23] as briefly outlined below. WHO advise that it is important to use assays with a minimum overlap of both false-positive and false-negative reactions. They recommend using both the Ortho 3.0 Enhanced SAVe (Ortho 3) (Ortho Clinical Diagnostics) and Monolisa anti-HCV Plus version 2 (Monolisa) (Bio-Rad; formerly Sanofi Diagnostics Pasteur) as initial screening assays. They further recommend that these screening assays are then confirmed by either the Chiron recombinant immunoblot assay (RIBA) or via nucleic acid testing (NAT) [22]. In addition, the use of signal-to-cut-off (S/CO) ratios are recommended, as these have been found to be a better predictor of sample status.

Alter et al. 2003 [20] reported that in a population where carriage is approximately 2% approximately 80% of the Ortho 3 reactive samples with a S/CO ratio of <3.8 were in fact negative, whilst approximately 97% of those reactive with a S/CO ratio greater than 3.8 were truly positive. They conclude that any samples with a S/CO ratio of less than 3.8 should be retested and anything greater than 3.8 can be considered positive. Similar evaluations of the Monolisa assay by Perry et. al., 2001 [24] indicated that only 5% of positive samples have a S/CO ratio less than 5. This is supported by Centres for Disease Control and Prevention Atlanta USA (CDC) and the Microbiological Diagnostics Assessment Service UK (MiDAS) [20], [43]. Their results suggest using S/CO ratio values to predict HCV-RNA positives, those that have been exposed but have cleared, and false-positive infections.

Owusu-Ofori et. al., 2005 [23] suggest an alternative, simpler protocol for resource-poor country screening. They recommend that samples reactive to two or more EIA should be considered positive for past or present infection.

Ortho 3.0 Enhanced SAVe (Ortho Clinical Diagnostics), and Monolisa anti-HCV Plus version 2 system (Bio-Rad formerly Sanofi Diagnostics Pasteur), EIA screening assays were carried out at the Division of Transfusion Medicine, Department of Haematology, Addenbrookes Hospital Cambridge in a two-step process. To identify putative positives, the sera were initially screened for HCV using the Ortho 3.0 Enhanced SAVe ELISA test system, (a qualitative enzyme-linked immunosorbent assay for the detection of antibody to HCV), as per the standard kit protocols. Any sample that tested reactive, or equivocally reactive, was then tested twice more. In step two, all 36 Ortho 3.0 reactive samples, as well as 150 randomly selected non-reactive (negative) samples, were then rescreened in triplicate with the Monolisa Anti-HCV Plus version 2 BioRad test system. S/CO ratios were calculated for all reactive samples. In our study, any sample that was positive for both the Ortho 3.0 and Monolisa tests was considered to be HCV antibody positive.

### Nucleic Acid Testing (NAT)

Next, viral RNA was extracted from all sera that tested reactive in either screening tests using Qiagen miniprep. Standard extraction protocols were used, with the modification that 500 µl of sera was first centrifuged at maximum rpm for 1 hour to concentrate the viral particles. From this, 360 µl of sera was pipetted off and RNA was then extracted from the remaining 140 µl. A 300 nt fragment of the NS5B and a 200 bp fragment of the 5′UTR regions were RT-PCR amplified as per the protocols outlined in Murphy *et. al.* 2007 [4] Table 4 provides the primer sequences for both regions. As the genotypes circulating in the region are unknown, primer combinations that would amplify all genotypes were used. RNA and DNA amplifications were conducted using both Superscript II for two-step amplifications, and Superscript III Platinum Taq for one-step amplifications (Invitrogen Life Sciences), followed by nested reactions with Roche Hifi Expand (Roche Diagnostics) using standard protocols. Controls were run in parallel at each step.

**Table 4:** Primers.

Primer	Primer sequences	Genotype
5′UTR Ex F1	CCCTGTGAGGAACT(AT)CTGTCTTCACGC	All
5′UTR In F3	TCTAGCCATGGCGTTAGT(AG)(CT)GAG	All
Core Ex R2	GGTGCACGGTCTACGAGACCT	All
Core In R4	CACTCGCAAGCACCCTATCAGGCAGT	All
NS5B Ex F	TGGGGATCCCGTATGATACCCGCTGCTTTGA	1–5 & ‘7’
NS5B Ex R	CGGAATTCCTGGTCATAGCCTCCGTGAA	1–5 & ‘7’
NS5B ExF G6	CCHATGGGGTTYTCCTAYGACAC	6
NS5B ExR G6	GGNGCYGAGTAYCTGGTCATGGC	6
NS5B In F	GACACCCGCTGCTTTGACTC	All
NS5B In R	GAGTCTTCACGGAGGCTATGACNAGGTA	All
860 Ex F	GCGCTTATCGCATACGGCTAG	rs12979860
860 Ex R	CCCAGCAGGCGCCTCTCCTA	rs12979860
860 In F	CCTGGACGTGGATGGGTACTG	rs12979860
860 In R	GCAGGCGCCTCTCCTATGTCAG	rs12979860
917 Ex F	CATACAACATGGAGAGTTAAAGTAAGTC	rs8099917
917 Ex R	GCTGGCCCCAGGAGCTTGCACTAG	rs8099917
917 in R	CCTGTGCTGGGCCACCACAATTCA	rs8099917

Primers used in RT-PCR, and PCR reactions of the 5′UTR and NS5B region of HCV and the nested amplifications of the IL28B SNPs. Genotype 7 is in quotations as it is yet to be confirmed as a new genotype. TaqMan SNP typing for rs8099917 was conducted using custom Assays-on-Demand products from Applied Biosystems (C__11710096_10) and verified with the listed primers, using standard PCR and sequencing techniques.

### IL28B typing

The confirmed HCV antibody positive participants (that is, sera samples positive in both serological assays) were haplotyped for both rs8099917 and rs12979860.

The rs8099917 haplotype was determined using TaqMan allelic discrimination with custom Assays-on-Demand products from Applied Biosystems (C__11710096_10) and confirmed by PCR reactions with Roche Hifi Expand polymerase (Roche Diagnostics). The rs12979860 haplotype was determined using nested PCR reactions with Roche Hifi Expand polymerase, standard protocols and Sanger sequencing. The primers used are listed in Table 4.

## Supporting Information

Table S1 **Ortho HCV 3.0, Monolisa Plus, HCV- NAT & IL28B SNP results.**
(DOCX)

Table S2 **Pacific Haplotypes.**
(DOCX)
