# Supplementary material for: Infection Frequency of Hepatitis C Virus and IL28B Haplotypes in Papua New Guinea, Fiji, and Kiribati
Source: PLoS One. 2013 Aug 20;8(8):e66749. doi: 10.1371/journal.pone.0066749 (PMC3748064; doi:10.1371/journal.pone.0066749)
Supplement: Table S1 — Ortho HCV 3.0, Monolisa Plus, HCV- NAT & IL28B SNP results. (DOCX) [file pone.0066749.s001.docx]

Supplementary Table S1: Ortho HCV 3.0, Monolisa Plus, HCV- NAT & IL28B SNP results

| Sample | Ortho HCV 3.0 screen | | | Ortho HCV 3.0 ratio values | | | Bio Rad screen | | | Bio Rad screen ratio values | | | NAT | | rs12979860 | rs8099917 |
| --- | --- | --- | --- | --- | --- | --- | --- | --- | --- | --- | --- | --- | --- | --- | --- | --- |
|  |  |  |  |  |  |  |  |  |  |  |  |  |  |  |  |  |
|  | 1 | 2 | 3 | 1 | 2 | 3 | 1 | 2 | 3 | 1 | 2 | 3 | 1 | 2 | CvT | TvG |
| P_054 | + | *nt* | *nt* | 2.97 |  |  | + | *nt* | *nt* | 2.72 |  |  | - | - | C | T |
| PD083 | + | *nt* | *nt* | 2.63 |  |  | *nt* | *nt* | *nt* |  |  |  | - | - |  |  |
| PW013 | + | *nt* | *nt* | 1.41 |  |  | + | *nt* | *nt* | 1.05 |  |  | - | - | C | T |
| PW017 | + | *nt* | *nt* | 1.38 |  |  | + | *nt* | *nt* | 1.13 |  |  | - | - | C | T |
| PW043 | + | *nt* | *nt* | 1.66 |  |  | *nt* | *nt* | *nt* |  |  |  | - | - |  |  |
| PW057 | + | *nt* | *nt* | 3.27 |  |  | - | *nt* | *nt* | 0.66 |  |  | - | - |  |  |
| PW062 | + | *nt* | *nt* | 1.39 |  |  | + | *nt* | *nt* | 1.03 |  |  | - | - | C | T |
| PW064 | + | *nt* | *nt* | 4.47 |  |  | + | *nt* | *nt* | 4.66 |  |  | - | - | C | T |
| PW085 | + | *nt* | *nt* | 2.32 |  |  | + | *nt* | *nt* | 2.32 |  |  | - | - | C | T |
| K_004 | + | - | - | 1.24 | 0.89 | 0.99 | - | - | - | 0.24 | 0.31 | 0.25 | - | - |  |  |
| K_063 | + | - | - | over | 0.47 | 0.14 | - | - | - | 0.66 | 0.01 | 0.21 | - | - |  |  |
| K_078 | + | - | + | 1.85 | 0.34 | 1.03 | - | - | - | 0.23 | 0.35 | 0.31 | - | - |  |  |
| K_084 | + | - | + | over | 0.67 | 1.04 | - | - | - | 0.63 | 0.22 | 0.21 | - | - |  |  |
| FA212 | + | + | + | 1.92 | 4.39 | 3.08 | - | - | - | 0.69 | 0.58 | 0.84 | - | - |  |  |
| F_004 | + | + | - | 1.19 | 1.17 | 0.43 | - | - | - | 0.43 | 0.18 | 0.29 | - | - |  |  |
| F_012 | + | + | + | 1.20 | 1.28 | 1.16 | - | - | - | 0.49 | 0.39 | 0.35 | - | - |  |  |
| F_013 | + | + | - | 1.91 | 1.04 | 0.00 | - | - | - | 0.60 | 0.49 | 0.58 | - | - |  |  |
| F_064 | + | - | - | 1.07 | 0.29 | 0.79 | - | - | - | 0.47 | 0.66 | 0.61 | - | - |  |  |
| F_120 | + | + | + | 2.06 | 1.47 | 1.24 | + | + | + | 3.16 | 3.05 | 2.19 | - | - | C | T |
| F_139 | - | + | - | 1.01 | 1.07 | 0.57 | - | - | - | 0.30 | 0.19 | 0.43 | - | - |  |  |
| F_167 | + | + | + | 1.48 | 2.73 | 2.19 | - | - | - | 0.59 | 0.98 | 0.84 | - | - |  |  |
| F_315 | + | + | + | 2.93 | 4.13 | 3.12 | - | - | - | 0.14 | 0.22 | 0.47 | - | - |  |  |
| P_023 | + | + | + | 2.35 | over | 3.65 | - | - | - | 0.27 | 0.22 | 0.37 | - | - |  |  |
| P_035 | + | + | + | 1.41 | 1.31 | 1.60 | + | + | + | 1.58 | 1.46 | 1.27 | - | - | C | T |
| P_039 | + | + | + | 3.31 | 2.11 | 3.28 | - | - | - | 0.28 | 0.30 | 0.40 | - | - |  |  |
| P_047 | + | + | + | 4.12 | 4.49 | 4.42 | + | + | + | 2.35 | 3.80 | 3.35 | - | - | C | T |
| P_109 | + | + | + | 1.64 | 4.63 | 2.04 | + | + | + | 5.28 | 6.89 | 7.38 | - | - | C | T |
| P_130 | + | + | + | 4.28 | over | 4.54 | + | + | + | 4.32 | 5.55 | 5.42 | - | - | C | T |
| P_172 | + | + | + | 2.11 | 2.25 | 4.23 | - | - | - | 0.57 | 0.22 | 0.22 | - | - |  |  |
| P_242 | + | + | + | 3.61 | over | 4.37 | + | + | + | 1.21 | 1.20 | 1.15 | - | - | Y | T |
| P_328 | - | + | + | 0.99 | 2.53 | 2.11 | + | + | + | 2.00 | 1.32 | 1.25 | - | - | C | T |
| PGM016 | + | + | + | 1.28 | 4.11 | 3.05 | + | + | + | 1.82 | 2.01 | 2.24 | - | - | C | T |
| PH080 | + | + | + | 1.26 | 1.39 | 1.63 | - | - | - | 0.33 | 0.13 | 0.18 | - | - |  |  |
| PH078 | + | + | + | 1.17 | 1.48 | 1.13 | - | - | - | 0.51 | 0.24 | 0.29 | - | - |  |  |
| PW018 | + | + | + | 4.33 | over | 5.23 | - | - | - | 0.70 | 0.38 | 0.33 | - | - |  |  |
| PW019 | + | + | + | 2.50 | 3.80 | 3.76 | - | - | - | 0.64 | 0.62 | 0.78 | - | - |  |  |
| PW023 | + | + | + | 1.07 | 2.84 | 2.33 | + | - | - | 1.34 | 0.23 | 0.34 | - | - | C | T |
| PW041 | + | + | + | 1.34 | 4.27 | 3.66 | - | - | - | 0.56 | 0.36 | 0.42 | - | - |  |  |
| **2008 Repeats** | | | | | | | | | | | | | | | | |
| PGM016 | - | - | *nt* | 0.60 | 0.40 |  |  |  |  |  |  |  | - | - |  |  |
| PW013 | + | + | *nt* | 3.44 | 3.94 |  |  |  |  |  |  |  | - | - |  |  |
| PW017 | + | + | *nt* | 1.76 | 2.40 |  |  |  |  |  |  |  | - | - |  |  |

This table details the participants who were identified as serologically reactive or positive for HCV antibodies and their corresponding HCV NAT results as well as their IL28B SNP haplotype. Serological cut-off ratios are given for each test conducted, in respective columns. “over” indicates samples that gave a positive result beyond the absorbance range. All samples were tested in triplicate, except those with limited serum available. The three repeat samples given at the bottom are those that were retested in the follow-up visit; they remained serologically reactive and HCV-RNA negative. Only the 15 confirmed HCV antibody positive participants were IL28B SNP-typed.
