# Supplementary material for: Infection Frequency of Hepatitis C Virus and IL28B Haplotypes in Papua New Guinea, Fiji, and Kiribati
Source: PLoS One. 2013 Aug 20;8(8):e66749. doi: 10.1371/journal.pone.0066749 (PMC3748064; doi:10.1371/journal.pone.0066749)
Supplement: Table S2 — Pacific Haplotypes. (DOCX) [file pone.0066749.s002.docx]

Supplementary Table S2: Pacific Haplotypes

| rs8099917 | Fiji Islands | Kiribati | Papua New Guinea |
| --- | --- | --- | --- |
| TT | 20 | 47 | 19 |
| GT | 0 | 1 | 1 |
| GG | 0 | 0 | 0 |
| Total samples | 20 | 48 | 20 |

88 individuals from Fiji, Kiribati, and Papua New Guinea were haplotyped for rs8099917, 86 of the 88 were found to be homogeneous for T - the major allele shown to be protective for HCV. The remaining 2 were heterogenetic (TG). 16 of the 20 Fijian Island individuals were indigenous Fijian the remaining 4 were, mixed Rotuman/Indian, Fijian/English, I-Kiribati, and Fijian/Tongan. All the I-Kiribati and Papua New Guinea samples were of indigenous ethnicity.
